# Trauma-related emotions and radical acceptance in dialectical behavior therapy for posttraumatic stress disorder after childhood sexual abuse

**DOI:** 10.1186/s40479-017-0065-5

**Published:** 2017-07-13

**Authors:** Nora Görg, Kathlen Priebe, Jan R. Böhnke, Regina Steil, Anne S. Dyer, Nikolaus Kleindienst

**Affiliations:** 1 Institute of Psychiatric and Psychosomatic Psychotherapy, Central Institute of Mental Health Mannheim, J5, 68159 Mannheim, Germany; 2 Medical Faculty Mannheim, Heidelberg University, Heidelberg, Germany; 3 Mental Health and Addiction Research Group, Hull York Medical School and Department of Health Sciences, University of York, York, YO10 5DD UK; 4 Department of Clinical Psychology and Psychotherapy, Institute of Psychology, Goethe University Frankfurt, Varrentrappstr. 40-42, 60486 Frankfurt am Main, Germany; 5 Institute of Cognitive and Clinical Neuroscience, Central Institute of Mental Health Mannheim, J5, 68159 Mannheim, Germany

**Keywords:** Posttraumatic stress disorder, Exposure therapy, Dialectical behavior therapy

## Abstract

**Background:**

Posttraumatic Stress Disorder (PTSD) related to childhood sexual abuse (CSA) is often associated with a wide range of trauma-related aversive emotions such as fear, disgust, sadness, shame, guilt, and anger. Intense experience of aversive emotions in particular has been linked to higher psychopathology in trauma survivors. Most established psychosocial treatments aim to reduce avoidance of trauma-related memories and associated emotions. Interventions based on Dialectical Behavior Therapy (DBT) also foster radical acceptance of the traumatic event.

**Methods:**

This study compares individual ratings of trauma-related emotions and radical acceptance between the start and the end of DBT for PTSD (DBT-PTSD) related to CSA. We expected a decrease in trauma-related emotions and an increase in acceptance. In addition, we tested whether therapy response according to the Clinician Administered PTSD-Scale (CAPS) for the DSM-IV was associated with changes in trauma-related emotions and acceptance. The data was collected within a randomized controlled trial testing the efficacy of DBT-PTSD, and a subsample of 23 women was included in this secondary data analysis.

**Results:**

In a multilevel model, shame, guilt, disgust, distress, and fear decreased significantly from the start to the end of the therapy whereas radical acceptance increased. Therapy response measured with the CAPS was associated with change in trauma-related emotions.

**Conclusions:**

Trauma-related emotions and radical acceptance showed significant changes from the start to the end of DBT-PTSD. Future studies with larger sample sizes and control group designs are needed to test whether these changes are due to the treatment.

**Trial registration:**

ClinicalTrials.gov, number NCT00481000

**Electronic supplementary material:**

The online version of this article (doi:10.1186/s40479-017-0065-5) contains supplementary material, which is available to authorized users.

## Background

Patients with Posttraumatic Stress Disorder (PTSD) typically report a wide range of aversive emotions (e. g., fear, disgust, sadness, shame, guilt, and anger) as well as heightened levels of affective instability [1–3]. An intense experience of aversive emotions has been linked to higher psychopathology in trauma survivors [4–14]. To emphasize the emotional consequences of traumatic experiences, the DSM-5 introduced two new criteria for PTSD as part of the new symptom cluster D “negative alterations in cognitions and mood” [15]: “Persistent, distorted cognitions about the cause or consequences of the traumatic event(s) that lead the individual to blame himself/herself or others” as well as “persistent negative emotional state (e.g., fear, horror, anger, guilt, or shame)”. These criteria extend the three PTSD symptom clusters that were previously defined in the DSM-IV-TR [16] and earlier versions (re-experiencing, avoidance/emotional numbing, and hyperarousal), as well as the central affective symptoms of restricted affect, distress during confrontation with trauma triggers, and irritability/outbursts of anger.

Trauma-focused treatments have shown to be efficacious for PTSD [17]. They reduce avoidance of memories and associated emotions. Research on affective changes in trauma-focused therapy has focused primarily on fear and non-specific distress-partly as a consequence of Foa and Kozak’s influential Emotional Processing Theory [18]. Within this framework, a pathological “fear structure” is defined as the central component of anxiety disorders and PTSD [19]. The framework posits that the reduction of fear and distress over the course of several exposure sessions (between-session) leads to reduced expectations of threat and subsequently to a change in the fear structure. Consequently, the between-session changes in self-reported fear and distress were hypothesized to be important process variables.

However, emotional consequences of trauma can differ widely between patients. In a pilot study by Power and Fyvie [20], about half of the 75 patients with mixed trauma types reported fear as the most prevalent emotion since the traumatic event. The other half reported a primary experience of disgust, sadness or anger that was associated with longer periods since the onset of psychological problems. Patients with interpersonal violence exposure (IPV) in particular reported elevated ratings of shame, guilt, fear, disgust, and anger in several studies [1, 2, 21]. Thus, focusing on emotions other than fear might be particularly relevant in studies on IPV-related PTSD [22, 23].

Studies showed that fear, shame, guilt, sadness, anger, and disgust significantly decrease from the start to the end of trauma-focused therapy [22, 24–28]. To date, a number of studies have investigated the link between PTSD symptomatology according to the DSM and fear or distress experienced within trauma-focused therapy [26, 29–36]. A recent meta-analysis showed that a between-session decrease in fear and distress is associated with a decrease of PTSD symptoms as defined by the DSM [37]. However, only a few studies have focused on links between PTSD symptomatology and other trauma-related emotions in trauma-focused therapy. In one study on women with IPV-related PTSD, a higher between-session decrease in sadness and anger was associated with remission after exposure therapy [26]. In that study, remission was defined according to the PTSD Symptom Scale-Interview (PSS-I) [38], and emotions were assessed during sessions. Similarly, another study measured PTSD symptomatology (re-experiencing, avoidance, and dissociation) as well as trauma-related emotions during a repeated imagery rescripting task for women with sexual assault experience. As a result, a between-session decrease in disgust was predictive of reduced PTSD symptomatology during the task but only in women who showed a significant between-session decrease in fear [39]. In contrast, a study of combat veterans did not find any statistically significant correlations between sadness, anger, and guilt as experienced during imaginal flooding sessions and the number of daily intrusions after therapy [28].

In other studies, emotions were not assessed in therapy sessions, but were assessed in other settings independent of therapeutic interventions. In one of these studies, patients with mixed trauma types received trauma-focused therapy and rated weekly levels of trauma-related shame and guilt [40]. Weekly changes in both emotions were positively correlated with subsequent changes in the PTSD Symptom Scale – Self-Rating (PSS-SR) [38]. Similarly, reductions in guilt from pre to mid treatment predicted reductions in the Clinician-Administered PTSD Scale (CAPS) [41] in a study with trauma-focused therapy for patients with IPV-related PTSD [24]. A study on psychotherapy for patients with PTSD related to childhood sexual abuse (CSA) who were at risk for Human Immunodeficiency Virus showed conflicting findings [25]: Pre-post-therapy reductions in shame, but not in guilt, correlated significantly with reductions in the Posttraumatic Stress Disorder Checklist–Specific (PCL-S) [42]. Overall, empirical data suggest that the between-session decrease in fear and distress is a potential proxy for changes in PTSD symptomatology as defined by the DSM-IV and earlier versions. However, the question of whether other trauma-related emotions are similarly relevant requires further investigation. To date, only a few studies [26, 28] have assessed a wide range of trauma-related emotions rather than only one or two specific emotions [25, 27, 40].

Another PTSD symptom cluster is avoidance and emotional numbing [15]. A recent meta-analysis linked the tendency to avoid painful emotions, thoughts, and memories (“experiential avoidance”) [43] to the severity of PTSD symptoms in samples with various trauma types [44]. “Third wave therapies” such as Acceptance and Commitment Therapy (ACT) [45] or Dialectical Behavior Therapy (DBT) [46] stress the importance of accepting and tolerating aversive emotions. For example, DBT teaches the concept of “radical acceptance”, which involves the acceptance of unchangeable emotions, thoughts, and unchangeable circumstances [46]. Steil and colleagues [47] combined elements of DBT with trauma-focused cognitive interventions and exposure therapy for patients with PTSD after CSA (DBT-PTSD) [48–51]. Following the DBT concept of radical acceptance, DBT-PTSD encourages patients to accept past traumatic events, painful memories of those events, and emotions about having experienced such adversities (instead of avoiding, rejecting and fighting). Some empirical evidence on the importance of acceptance comes from ACT for patients with chronic pain where acceptance of pain mediated the treatment effect on physical functioning [52]. To our knowledge, no previous empirical study has yet examined the pre to post change of radical acceptance in DBT. Given the central role that radical acceptance plays in DBT-based treatments, it would be clinically relevant to test whether this variable is subject to change.

### Research questions

In summary, some empirical evidence has shown that trauma-related emotions decline between the start and the end of trauma-focused treatments. In addition, higher decreases in fear and distress between therapy sessions were linked to higher decreases in PTSD symptomatology according to the DSM-IV and earlier versions. However, research about the link between PTSD symptomatology and trauma-related emotions beyond fear is limited. It also remains unclear whether radical acceptance according to DBT definitions changes from the start to the end of DBT-based trauma-focused therapy. This study investigates the change in trauma-related emotions and radical acceptance from the start to the end of DBT-PTSD. We hypothesized that there would be a decrease in all negative trauma-related emotions and an increase in radical acceptance over time. Furthermore, the study aims to replicate the well-established links between distress, fear, and PTSD symptomatology. Potential links between other trauma-related emotions, radical acceptance and PTSD symptomatology according to the CAPS [41] are also explored. The data were collected within a subsample of a randomized controlled trial (RCT) which tested the efficacy of DBT-PTSD. In the original study, DBT-PTSD was found to be superior to a treatment-as-usual waitlist control group (TAU) with large effect sizes in a self-reported and clinician-administered PTSD measure. The main results were published elsewhere [48]. Here, only data from patients receiving DBT-PTSD was analyzed.

## Methods

### Sample

Female participants aged 17 to 65 years old with a current diagnosis of PTSD related to CSA were included in the RCT [48]. In addition, at least one of the following criteria had to be met: meeting four or more DSM-IV criteria of borderline personality disorder (BPD), current eating disorder, current major depressive disorder, or current substance abuse. While PTSD in trauma-exposed samples with a history of CSA is frequently accompanied by comorbidities such as substance abuse, alcohol abuse, or BPD [53], patients with such comorbidities as well as eating disorders or increased suicide risk are often excluded from studies [54–57]. To increase the external validity these comorbidities were included in the original RCT. Exclusion criteria were: medical contraindications for exposure treatment (e.g., severe cardiovascular disorders; body mass index <16.5), life-threatening behavior within 4 months prior to study entry, intellectual disability, a lifetime diagnosis of schizophrenia or bipolar I disorder, or a current diagnosis of substance dependence.

Within the RCT, patients were randomized to receive either DBT-PTSD or the TAU. In the DBT-PTSD group, 39 patients started the therapy. After the study period, all patients from the TAU group (*n* = 39) were offered DBT-PTSD treatment and 32 of the 39 patients started the treatment. To increase the sample size, this analysis included both patients from the original DBT-PTSD trial arm as well as patients from the TAU group if they received DBT-PTSD after the original study period. Only data collected during the DBT-PTSD treatment were included. Ratings of emotions and acceptance were introduced at a later stage of the study period, so that data on trauma-related emotions would be available for a subsample. Our analysis required at least two assessments of trauma-related emotions during the start (week 2–4) and end (final two consecutive weeks before discharge) of therapy. These data were available for 28 patients, and 23 patients completed the diagnostic sessions at the beginning and end of therapy. Within the final sample of 23 patients, 15 patients came from the DBT-PTSD group and 8 patients were originally in the TAU group and eventually received the active treatment.

### Treatment

Participants received between 12 and 14 weeks of a modular residential treatment at the PTSD unit of the Central Institute for Mental Health, Mannheim, Germany (CIMH). The detailed treatment protocol of this study is described elsewhere [48]. Week one to week four mainly included psychoeducation of PTSD: teaching of DBT skills and identification of individual avoidance behavior (e. g. dissociation, self-harm, and cognitive denial). Patients received imaginal exposure from week five to week 10. Between the sessions, patients listened to audio-recordings of the exposure sessions as a self-administered exposure exercise. During exposure, DBT interventions (e.g., distraction skills) could be used to ensure awareness of the present as opposed to dissociative states or flashbacks. Furthermore, emotion regulation strategies could be applied to down-regulate overwhelming emotional responses. In addition, there were cognitive interventions that focused on guilt and discrimination between the current and the traumatic situation [58]. In the final 2 weeks, specific interventions aimed at achieving radical acceptance. Patients received biweekly psychotherapy sessions and took part in several group activities (11 sessions of 90 min DBT skills training, eight 60 min sessions of skills training for self-esteem, 35 sessions of 25 min mindfulness training, 11 sessions of 60 min psychoeducation on PTSD and weekly group interventions on music or art therapy). The therapy was delivered by clinical psychologists with additional training in DBT and trauma-focused therapy. Participants in the TAU-WL group were allowed to seek any kind of treatment except for DBT-PTSD within the 6-month study period.

### Assessments

Diagnosis of PTSD following CSA and axis I comorbidities were checked with the Structured Clinical Interview for DSM-IV Axis I Disorders [59]. BPD symptoms were diagnosed with the International Personality Disorder Examination (IPDE) [60]. The outcome measure used in this study was the CAPS [41]. Ratings referred to the index event, i.e., the traumatic situation that is currently causing the highest level of distress. Global psychopathology was assessed with the Symptom Checklist 90-R (SCL-90-R) to compute the Global Severity Index (GSI) [61]. The CAPS was assessed before and after DBT-PTSD treatment. Ratings on trauma-related emotions were filled out directly before treatment sessions. Originally, these assessments served as a feedback instrument to measure the patients’ progress regarding trauma-related emotionality. It was not designed for study purposes. In the questionnaire, patients were asked to think of the index event and then rate their levels of shame, guilt, distress, disgust, fear, anger, sadness, and radical acceptance in response to it. The scale ranged from 0 (not at all) to 100 (maximum). Psychoeducation in all trauma-related emotions and radical acceptance was offered in the skills groups of the treatment.

### Statistical analyses

**Fig. 1:**
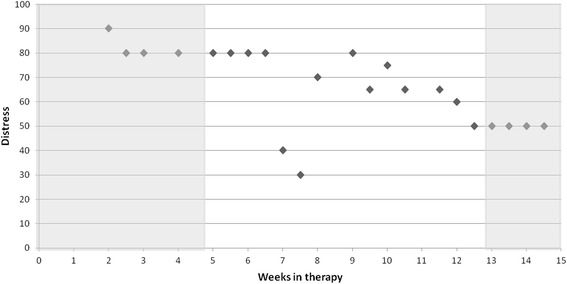
Illustration of data inclusion: Change of distress ratings of a participant. Sessions within weeks 2–4 were used to calculate emotion scores at the start of the treatment. The end of the treatment comprised the final 2 weeks before admission (weeks 13 and 14). Only sessions marked in grey were used to estimate the models

To test which emotions were predominant at the start (week 2–4) of the treatment, eight two-sided *t*-tests with a Bonferoni corrected Alpha level of *α* = .006 were computed. Each *t*-test compared scores for one variable with the average of all other variables (emotions and acceptance). To investigate whether trauma-related emotions decreased (and acceptance increased) over time, we tested whether these assessments changed on average between the start (week 2–4) and the end (final 2 weeks) of therapy. This was done on a descriptive level and with multilevel models (MLM). Next, we tested whether treatment outcome as assessed with the CAPS had an incremental effect on predicting trauma-related emotions and acceptance. For each treatment phase (start vs. end), at least two and up to seven assessments of trauma-related emotions and acceptance per patient were available (see Fig. 1). The MLM used repeated data that were nested within patients.

Four models for each emotion and acceptance were computed. In model 1, we estimated the intra-class correlations (ICCs) for these data without the information whether the rating was at the start or at the end of the treatment. This quantifies the amount of observed differences between patients, and it serves as a baseline model to test whether adding predictors significantly increases model fit.

In model 2, we added the treatment phase (0 = start; week 2–4 vs. 1 = end of treatment, final 2 weeks before discharge) as a fixed effect on the level of the patient. According to the DBT-PTSD protocol, these two treatment phases correspond with the pre- and post-exposure phase. This fixed effect therefore captures the average difference between treatment phases across all patients.

In models 3 and 4, we tested whether treatment outcome had an incremental effect on trauma-related emotions and acceptance. Treatment outcome was either included as a dichotomous (model 3) or as a continuous predictor (model 4). In model 3, we included whether the patient responded to the therapy or not; (“response”) as a dichotomous predictor on the between-patient level. “Response” was defined as a reduction in CAPS scores of at least 30 from the start to the end of the treatment [48, 62]. In model 4, we used the reduction in the CAPS scores from the start to the end of the treatment as a continuous predictor on the between-patient level. Both were added as fixed effects to the model. Patients were included as a random effect in all models. Further details on the MLMs can be found in Additional file 1.

To choose the model with the best fit to the data we used the corrected Akaike information criterion (AICc) which has been shown to be more appropriate in smaller samples-especially in models for longitudinal data [63, 64]. Lower values indicate a better fit. We evaluated whether the inclusion of treatment phase as a predictor increased model fit relative to a non-trend model when predicting trauma-related emotions and acceptance (comparison between model 2 and model 1). We also evaluated whether including therapy outcome as a predictor had an incremental effect on model fit (comparison between model 3 and model 2 and between model 4 and model 2). Furthermore, *R* ^2^ was computed to illustrate the fit of the models to the data. This represents the squared correlation between the observed values and the predicted values of each model based on the included fixed effects. The weight of evidence *(W)* was computed to illustrate the probability that a model provides the best fit when compared with the three other models [63]. *W* states how likely each model is the best available approximation of the data compared to the other available models. For graphs and descriptives we used IBM SPSS Statistics 21; MLM analysis were done with the R software version 3.1.3 [65], package lme4 [66].

## Results

### Sample characteristics

The average age of the all-female sample was 36.3 (*SD* = 10.5; range 20 to 52 years). Patients initially had an average CAPS severity score of 88.1 (*SD* = 15.2) which was comparable to the original entire RCT sample (*M* = 85.2, *SD* = 16.38) [48]. The average GSI in our subsample was 1.99 (*SD* = 0.66) (entire sample: *M* = 1.95, *SD* = 0.62). Patients in our subsample showed an average decrease in CAPS scores of 32.0 (*SD* = 25.7). Of the 23 patients, 14 fulfilled the response criterion at the end of the therapy (reduction of at least 30 points reduction in the CAPS [42]). For responders, the average decrease in CAPS scores was 51.8 (*SD* = 19.2) and 10.8 for non-responders (*SD* = 9.6). Within this subsample, 12 patients (52%) fulfilled a diagnosis of BPD according to the IPDE compared to 45% in the entire RCT sample. On average, patients in our subsample fulfilled 4.3 BPD criteria (*SD* = 2.0) and 4.06 (*SD* = 1.88) in the entire sample. In this subsample, patients had an average of 2.78 axis I disorders compared to 3.01 axis I comorbidities across the entire sample. The most frequent comorbidity in both samples was major depression (subsample: 83%, entire sample: 80%). Altogether, 78% of patients in the subsample (86% in the entire sample) received psychotropic medication-most of them antidepressants (subsample and whole sample: 70%). A more detailed description of the entire RCT sample can be found in the main paper [48].

### Data description

**Fig. 2:**
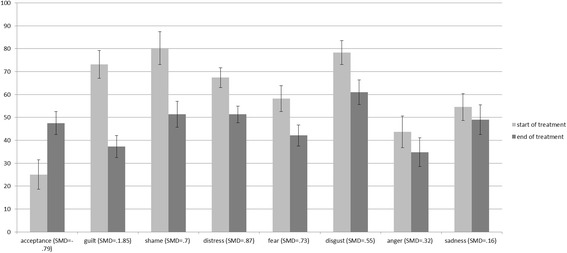
Change in trauma-related emotions and acceptance; Mean ± 1 SE of trauma-related emotions at the start and end of the treatment. In brackets: Standardized mean of the differences (SMD)

Six of the eight *t*-tests that compared one variable (emotion or acceptance) with the average score of all other variables at the start of the therapy were significant. Only the *t*-tests for fear and sadness were non-significant. In line with Power’s and Fyvie’s findings [20], patients did not report one predominant emotion at the start of the treatment, but showed heightened levels of different emotions. We illustrated whether a change in emotions resp. acceptance could be observed between the start (week 2–4) and the end (final 2 weeks before discharge) of therapy. Figure 2 shows that all of the trauma-related emotions decreased over time, whereas radical acceptance increased. This pattern of change is in line with our prior expectations.

### Multilevel modeling

MLMs for the prediction of each trauma-related emotion and acceptance were computed separately. Next, the fit of models was compared between model 1 (no trend), model 2 (inclusion of therapy phase as a predictor), model 3 (inclusion of therapy phase and response as predictors), and model 4 (inclusion of therapy phase and CAPS change as predictors) based on the AICc. The model parameters can be found in Table 1.

**Table 1:** Fit statistics for the different models for each emotion and acceptance. Model 3 operationalized therapy outcome as response (CAPS reduction of at least 30 points from the start to the end of the therapy vs. non-response). Model 4 operationalized therapy outcome as absolute reduction in CAPS scores from start to end

		Model 1 (no trend)	Model 2 (linear trend of therapy phase)	Model 3 (linear trend of therapy phase and response)	Model 4 (linear trend of therapy phase and CAPS change)
Guilt^a,b^	AICc	1729.90	1527.83	1522.38	1530.22
	W	0.00	0.06	0.92	0.02
	R^2^		.26	.26	.27
Shame	AICc	2010.35	1992.20	1987.85	1995.67
	W	0.00	0.10	0.88	0.02
	R^2^		.05	.05	.05
Distress	AICc	1620.93	1564.61	1557.53	1564.99
	W	0.00	0.03	0.95	0.02
	R^2^		.12	.16	.16
Fear	AICc	1658.39	1586.50	1577.68	1581.16
	W	0.00	0.01	0.84	0.15
	R^2^		.12	.19	.21
Anger	AICc	1640.97	1590.63	1583.36	1592.28
	W	0.00	0.03	0.96	0.01
	R^2^		.03	.09	.03
Sadness	AICc	1723.26	1661.58	1657.21	1665.10
	W	0.00	0.10	0.88	0.02
	R^2^		.01	.01	.01
Disgust	AICc	1711.20	1630.35	1625.92	1633.21
	W	0.00	0.10	0.88	0.02
	R^2^		.06	.06	.07
Acceptance	AICc	1631.22	1526.32	1521.56	1529.63
	W	0.00	0.08	0.90	0.02
	R^2^		.15	.16	.15

Note

^a^R^2^: correlation between observed and predicted values of each model

^b^W: Weight of evidence for model in the context of all other models

According to the AICc scores, model 1 showed the worst fit (highest AICc values) for each trauma-related emotion and acceptance. Thus, the models including the time in therapy were superior to the baseline models. The fixed effects were all in line with our hypotheses (that the intensity of negative emotions would decrease over time while acceptance would increase). When adding therapy response as a dichotomous predictor (model 3), the model fit increased further for every emotion and acceptance. When adding therapy response as a dimensional predictor (model 4), model fit increased only in the case of fear in comparison to model 2. However, in all cases, model 3 is the most parsimonous description of the data (lowest AICc).

The results are explained in detail for one emotion to illustrate the selection decisions. In the case of guilt, models 1 and 2 receive very low weights of evidence, indicating that adding response as a predictor (model 3) increases the fit to the data substantially. Model 3 is likely the most appropriate model compared to all other models.It has the lowest AICc score (1522.38) and the highest *W* (0.92) of all four models. This indicates that not only the inclusion of the response increases preditive power (compared to models 1 and 2), but that the inclusion of the dichotomous response provided better fit than the continuous CAPS score (model 4, *W* = .02). To conclude, the overall therapy outcome assessed with the independent criterion CAPS adds information only when used as a dichotomous predictor (response vs non-response)-not when used as a continuous predictor. The trends described for guilt are found for all variables and only for fear the dimensional predictor of therapy response (model 4) added some predictive value.

**Fig. 3:**
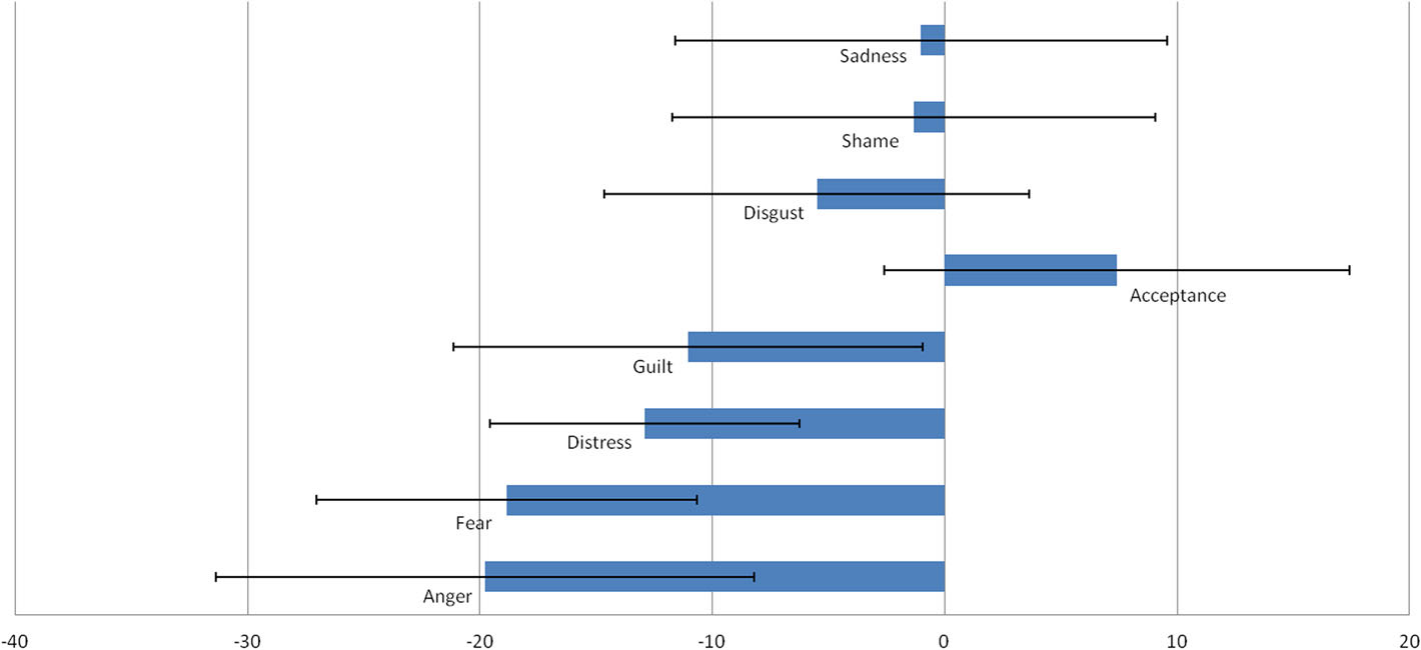
Model coefficients; Estimated coefficients ±1 SE for the effect of therapy response on trauma-related emotions

Table 2 presents the estimated fixed effects of model 3 for all emotions and acceptance. All estimates for the effect of treatment phase had expected trends, with decreases for emotions and increases of acceptance ratings. The estimated changes differ widely, from a decrease of 6.20 points in sadness to a 35.41 decrease in guilt. Similarly, the response in the CAPS correlates with a reduction in emotions between 1.01 points (sadness) and 18.85 points (fear). Due to the size of the sample, the standard errors of the individual effects are rather large and changes in anger and sadness over time are not statistically robust because their respective standard errors would lead to non-significant estimates (size of estimated coefficient compared to 1.96 x SE). For the association with CAPS response, only fear and perhaps distress can be seen as robust with regard to the significance of the individual predictors (see Fig. 3).

**Table 2:** Intercepts and slopes of models 3 estimated with the MLMs using time in therapy and therapy response (response vs non-response) to predict change in emotions and acceptance over time (fixed effects (standard error); scale: 0–100)^a,b^

	Estimated intercept (SE)	Coefficient Treatment phase (SE)	Coefficient Response (SE)
Guilt	79.90 (8.71)	−35.41 (4.03)	−11.05 (10.10)
Shame	80.85 (9.98)	−27.28 (9.10)	−1.34 (10.39)
Distress	75.20 (5.81)	−16.31 (3.83)	−12.91 (6.64)
Fear	69.92 (7.31)	−16.51 (4.55)	−18.85 (8.19)^a^
Anger	55.79 (9.90)	−9.40 (5.67)	−19.77 (11.59)
Sadness	55.64 (8.68)	−6.20 (6.97)	−1.01 (10.57)
Disgust	81.44 (7.73)	−16.45 (5.95)	−5.50 (9.15)
Acceptance	20.41 (8.91)	22.51 (5.66)	7.42 (10.02)

Note

^a^The estimated coefficient for CAPS as a continuous predictor for fear (best fitting modell according to AICc) was −16.54

^b^Time in therapy was coded as “0” (week 2–4 = start) or “1” (final 2 weeks = end treatment). The response was coded as “0” (CAPS change under 30) or response (CAPS change at least 30)

In a post hoc analysis, we additionally compared trauma-related emotions at three different time points: t0 (start of the treatment), t1 (2 weeks prior to discharge), and t2 (end of the treatment) via repeated measures *t*-tests and standardized means of the differences *(SMD).* The comparison between t1 and t2 corresponds with the start and the end of the acceptance-focused interventions. While guilt (*SMD* = −1.12) and shame (*SMD* = −0.72) declined significantly from t0 to t1, non-significant reductions were found in distress (*SMD* = −0.45), disgust (*SMD* = −0.34), sadness (*SMD* = −0.13), anger (*SMD* = −0.14), fear (*SMD* = −0.38) and non-significant increases in acceptance (*SMD* = 0.42). Non-significant reductions between t1 and t2 were found in guilt (*SMD* = −0.59), fear (*SMD* = −0.54), disgust (*SMD* = −0.50), shame (*SMD* = −0.35), distress (*SMD* = −0.34), sadness (*SMD* = −0.32), and anger (*SMD* = −0.03), whereas acceptance (*SMD* = 0.51) increased non-significantly. Thus, the variables changed in the expected direction in all treatment phases (start of treatment, start and end of acceptance-focused interventions).

## Discussion

This study investigated whether trauma-related emotions and radical acceptance changed from the start to the end of DBT-PTSD. Furthermore, the potential link between this change and the therapy response according to the Clinician-Administered PTSD Scale was explored [41]. Expanding upon previous studies, we not only investigated the role of fear and distress but also included other trauma-related emotions and radical acceptance. Overall, statistically parsimonious descriptions of the data suggest that patients experienced statistically significant reductions in shame, guilt, disgust, distress, and fear and increases in radical acceptance from the start to the end of the therapy. The model comparisons based on information criteria suggested that all trauma-related emotions and radical acceptance could potentially be correlated with a change in the CAPS according to DSM-IV. However, inferences on specific emotions should be made with caution due to the small sample size and standard errors.

Trauma-related emotions play a crucial role in the treatment of PTSD [22, 23]. Third wave therapies emphasize the importance of acceptance-based strategies to deal with unwanted thoughts, feelings and memories [45, 46]. DBT-PTSD aims at reducing a broad range of trauma-related emotions while fostering radical acceptance as a functional way to deal with traumatic memories. Along with previous studies [22, 24–28], this study found a decrease in a broad range of trauma-related emotions from the start to the end of trauma-focused therapy. Furthermore, the results showed that radical acceptance increased during DBT-PTSD. Future studies should use larger sample sizes and control group designs to test whether these changes can be attributed to a treatment effect. However, these results suggest that along with PTSD symptoms, a range of emotions and acceptance are subject to change in DBT-PTSD. While DBT-PTSD explicitly defines radical acceptance as a treatment target, other trauma-focused treatment focus on decreasing avoidance of trauma-related memories, emotions and thoughts. It should be tested in future studies whether trauma-related treatment per se is followed by increases in radical acceptance.

As a next step, the individual emotional profile of patients with PTSD could support differential indications. For example, CPT was superiour in decreasing trauma-related guilt in comparison to Prolonged Exposure [27]. Thus, CPT might be recommended for patients with heightened levels of trauma-related guilt. A promising approach could involve monitoring and feedback systems, which have been established in other areas of mental health for some time [67, 68]. In such systems, data are gathered continuously alongside treatment. These data can identify patients at risk of treatment failure [69]. Combining predictions from Emotional Processing Theory and emerging results such as ours, could establish assessments to guide treatment decisions regarding specific emotion-focused interventions [70]. In the original RCT on DBT-PTSD, more than 60% of the patients did not show a remission of PTSD symptoms 3 months after treatment [48]. Tailoring the treatment for specific trauma-related emotions could be one way to improve the overall treatment efficacy. While different studies have shown an association between changes in distress, fear, and PTSD symptomatology from the start to the end of trauma-focused treatment, results regarding other trauma-related emotions are mixed. The diverging results might be a consequence of differences in the operationalization of treatment outcome (dimensional vs. dichotomous), the context of the assessment (during exposure sessions or independent of the session), and trauma types (mixed trauma types, veterans, and IPV). Another possible explanation is that assessments such as the CAPS, PCL, PSS-I, or PSS-SR could be more closely related to fear and distress than to other emotions because they go back to the conceptualization of PTSD as an anxiety disorder. In concordance with that, a study on the contribution of global guilt, guilt cognitions, and distress on the prediction of PTSD symptomatology suggested that distress might be the strongest predictor [71]. Therefore, trauma-related shame, guilt, anger, sadness, and disgust could potentially represent pathognomonic aspects of PTSD symptomatology that have not been covered sufficiently by the CAPS. More specifically, emotions related to the self-concept such as shame, guilt, and disgust could potentially be a neglected area in earlier conceptualizations of PTSD [72, 73]. These emotions are related to different psychopathological symptoms: Suicidal ideation is associated with higher levels of guilt among military personnel [5] and with higher levels of sadness, guilt, and shame-proneness in women with major depression and a history of CSA, even after controlling for PTSD symptoms and other covariates [14]. Thus, trauma-related emotions might be important variables to assess during trauma-focused therapy in addition to standard PTSD measures.

### Strengths and limitations

The study has several strengths and limitations. One strength is that the study was performed within the relatively controlled environment of the RCT. The study used standardized diagnostic intake assessments, and different treatment phases comprised similar interventions for each patient due to the manualization of the therapy as well as intensive training and supervision of the study therapists. These factors contribute to the higher internal validity of our results. The small sample size is a clear limitation of our study and other studies on trauma-related emotions [26, 28]. However, the fact that we found significant effects within a limited number of study participants suggests relatively large effects for at least some of the dimensions. Therefore, future studies with larger sample sizes and different patient populations are needed when investigating trauma-related emotions.

Due to the limited sample size and high intercorrelations between the different emotions and acceptance, mediation analysis or testing of differential predictive power of individual emotions was not possible. However, this would be an important next step requiring larger sample sizes. Another limitation is that both the CAPS and the questionnaires on trauma-related emotions and acceptance focused on one index event. Thus, these measures could overestimate symptom improvement. It remains an open question whether PTSD symptomatology, trauma-related emotions and acceptance change only with regards to the index event, or whether this effect generalizes to other traumatic events.

Furthermore, each emotion was assessed with a single item to reduce the burden on the respondents, but this might limit the findings’ construct validity. The repeated short assessments across the course of the therapy still enabled reliable differentiation between patients as is evident in the ICCs. For the start of the therapy, ICCs in the step two models varied between 0.63 and 0.89 except for shame that had an ICC of 0.14. For the end phase of the treatment, ICCs ranged from 0.63 to 0.90. However, future studies should focus on the assessment of a few trauma-related emotions assessed via several items per emotion.

At first glance, our analytic strategy of using model-based averages of several assessments at the start and end of the therapy might seem limited. Nevertheless, as Fig. 1 shows, it is far from clear what kind of change should be assumed (when using growth models, for example [74]) or how to deal with the slightly different lengths of treatment in a fixed assessment mixed model analysis [75]. The chosen approach uses the individually defined treatment phases to derive a meaningful aggregate estimate of the treatment effect (Fig. 1). This increases the amount of data used in comparison to a pre-post repeated measurement ANOVA, and the use of all available data points in a MLM increases the reliability of start- and end-averages. Furthermore, by relying on differences between treatment phases this approach also uses a definition of change that stresses the importance of detecting differences between stretches of time, i.e., stable levels, instead of only single assessments [74, 76]. Finally, this study assessed only trauma-related emotions before therapy sessions. Future studies could increase the generalizability of the findings via ecological momentary assessments [77].

## Conclusion

This study is the first to our knowledge that has investigated changes in specific trauma-related emotions and radical acceptance within treatment for PTSD after CSA. This cohort showed a significant decline in trauma-related shame, guilt, disgust, distress, and fear. Due to the lack of a control group, it is unclear whether treatment induced these changes. Patients who showed a treatment response according to the CAPS had a stronger decrease in fear across both time points (start and end). The therapy response was related to decreases in all other trauma-related emotions and increases in acceptance, but this relationship did not reach statistical significance in most of the measures due to the small sample size and high standard errors. In conclusion, future studies with larger sample sizes are needed to assess the change in trauma-related emotions during trauma-focused therapy in addition to standard measures of PTSD symptomatology. We suggest testing and building feedback systems on trauma-related emotions. They could be used for the differential indication of emotion-specific interventions. Radical acceptance is an important yet rarely investigated variable in DBT-rooted exposure therapy for PTSD that increased from the start to the end of DBT-PTSD.

## Additional file

Additional file 1: Model notation: Notation for the four multilevel models used to test the main hypotheses. (DOCX 19 kb)

## Abbreviations

ACT: Acceptance and Commitment Therapy
AICc: corrected Akaike information criterion
CAPS: Clinician-Administered PTSD Scale
CIMH: Central Institute of Mental Health Mannheim (Germany)
CSA: Childhood sexual abuse
DBT: Dialectical Behavior Therapy
DBT-PTSD: Dialectical Behavior Therapy for Posttraumatic Stress Disorder
ICC: Intra-class correlation
IPDE: International Personality Disorder Examination
IPV: Interpersonal violence
MLM: Multilevel model
PCL-S: Posttraumatic Stress Disorder Checklist–Specific
PSS-I: PTSD Symptom Scale – Interview
PSS-SR: PTSD Symptom Scale – Self-Rating
PTSD: Posttraumatic Stress Disorder
SMD: Standardized mean of the differences
