# Supplementary material for: Trauma-related emotions and radical acceptance in dialectical behavior therapy for posttraumatic stress disorder after childhood sexual abuse
Source: Borderline Personal Disord Emot Dysregul. 2017 Jul 13;4:15. doi: 10.1186/s40479-017-0065-5 (PMC5508787; doi:10.1186/s40479-017-0065-5)
Supplement: Additional file 1: — Model notation: Notation for the four multilevel models used to test the main hypotheses. (DOCX 19 kb) [file 40479_2017_65_MOESM1_ESM.docx]

Additional File to Görg, N., Priebe, K., Böhnke, J.R., Steil, R., Dyer, A.S. & Kleindienst, N. Trauma-related Emotions and Radical Acceptance in Dialectical Behavior Therapy for Posttraumatic Stress Disorder after Childhood Sexual Abuse. *Borderline Personality Disorder and Emotion Dysregulation*, accepted.

**Additional file: Model notation**

Notation for the four multilevel models used to test the main hypotheses.

The overall structure is that several observations (*i* ; up to seven, see Figure 1) are nested within *j* patients. In model 1 the dependent variable is either a score of trauma-related emotions or acceptance. It is predicted by a patient-specific intercept (*β*_0j_), which again is predicted by an overall mean across all patients (γ_00_). The two error terms of this model (*ε*_ij_ patient-specific residual; *τ*_00_ variance of patients' mean levels around the overall mean) can be used to estimate the intraclass correlation.

In model 2, the treatment phase of the individual assessment is used as a within-patient predictor (0 = start of treatment; 1 = end of treatment). The corresponding fixed effect γ_10_ estimates the overall effect of time in treatment.

In models 3 and 4, the change in CAPS was added either as a dichotomized predictor (model 3 in text) or as a continuous change variable (model 4 in text). In either case, the corresponding fixed effect of γ_01_ provides the information of how much of an effect between-patient differences in attaining the treatment goal had above and beyond individual variation in change (captured in model 2 and by *β*_1j_ in this model).

Model 1:

Eq. (A.1): y_ij_ = β_oj_ + ε_ij_

Eq. (A.2): β_0j_ = γ_00_ + τ_00_

Model 2:

Eq. (B.1): y_ij_ = β_0j_ + β_lj_late + ε_ij_

Eq. (B.2): β_0j_ = γ_00_ + τ_00_

Eq. (B.3): β_lj_ = γ_10_ + τ_10_

Model 3/ Model 4:

Eq. (C.1): y_ij_ = β_0j_ + β_lj_late + ε_ij_

Eq. (C.2): β_0j_ = γ_00_ + γ_01_CAPS + τ_00_

Eq. (C.3): β_lj_ = γ_10_ + τ_10_
